# Bifenthrin resistance in *Dalbulus maidis* (Hemiptera: Cicadellidae): inheritance, cross‐resistance, and stability

**DOI:** 10.1002/ps.8848

**Published:** 2025-04-25

**Authors:** Gabriel Silva Dias, Eduardo Perkovski Machado, Matheus Gerage Sacilotto, Leonardo Vinicius Thiesen, Celso Omoto

**Affiliations:** ^1^ Department of Entomology and Acarology, Luiz de Queiroz College of Agriculture University of São Paulo Piracicaba Brazil

**Keywords:** corn leafhopper, insect resistance management, pyrethroid, stability of resistance

## Abstract

**BACKGROUND:**

Pyrethroid insecticides have been a primary strategy for managing *Dalbulus maidis* (Hemiptera: Cicadellidae) in Brazil. Howeve, failures in the control of *D. maidis* with pyrethroids have been reported. In this study, we selected a bifenthrin‐resistant strain of *D. maidis* under laboratory cage conditions to investigate the inheritance pattern of resistance, cross‐resistance to other insecticides, and resistance stability.

**RESULTS:**

The estimated LC_50_ of the Bif‐R was 2,055.72 μg a.i. mL^−1^, while that of the susceptible (Sus) strain was 0.64 μg a.i. mL^−1^, resulting in a 3,170‐fold resistance ratio (RR). Reciprocal crosses (H1: Bif‐R ♀ × Sus ♂ and H2: Bif‐R ♂ × Sus ♀) and backcrosses between heterozygous H1 and H2 with the Sus strain indicated autosomal, incompletely dominant, and polygenic resistance. Potential cross or multiple‐resistance was observed between Bif‐R and lambda‐cyhalothrin, imidacloprid, and acetamiprid, with resistance ratios varying from 300‐ to 2,000‐fold. No cross‐resistance was detected between Bif‐R and methomyl, carbosulfan or acephate. Cage studies with different proportions of Sus and Bif‐R strains revealed that resistance of *D. maidis* to bifenthrin is unstable. A decrease in the LC_50_ of the field‐collected population from 113.61 to 10.73 μg bifenthrin mL^−1^ was detected in the absence of selection pressure.

**CONCLUSIONS:**

Our findings provide insights into the evolution of resistance of *D. maidis* to bifenthrin. This study is the first comprehensive analysis of pyrethroid resistance in *D. maidis* and will contribute to insect resistance management (IRM) strategies to preserve the efficacy of bifenthrin and other insecticides. © 2025 The Author(s). *Pest Management Science* published by John Wiley & Sons Ltd on behalf of Society of Chemical Industry.

## Graphical abstract

Resistance of *Dalbulus maidis* to bifenthrin was autosomal, incompletely dominant, and polygenic. Additionally, this resistance was unstable in the absence of selection pressure.

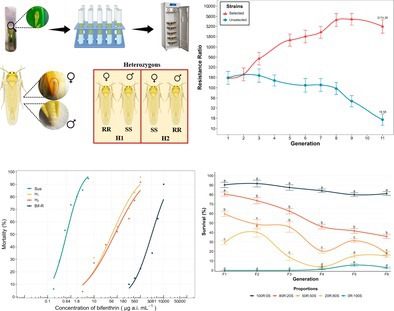

## 1 INTRODUCTION

The corn leafhopper, *Dalbulus maidis* (DeLong & Wolcott, 1923) (Hemiptera: Cicadellidae), is a vector of the pathogens that cause the corn stunt disease complex, including Corn Stunt Spiroplasma (CSS), Maize Bushy Stunt Phytoplasma (MBSP), Maize Rayado Fino Virus (MRFV) and Maize Striate Mosaic Virus (MSMV).^1^, ^2^, ^3^ The recent increase in *D. maidis* occurrence across various regions of Brazil is likely due to the intensification of the corn production, with corn plants present in fields year‐round either as crop or volunteer plants, the use of more susceptible varieties, favorable climatic conditions, and the evolution of resistance to insecticides.^4^, ^5^, ^6^, ^7^, ^8^, ^9^, ^10^, ^11^, ^12^, ^13^ Furthermore, studies have shown that *D. maidis* can feed and survive on alternative hosts,^8^ supporting population growth in the field. This combination of factors has ensured a steady food supply, creating favorable conditions for population outbreaks. Currently, volunteer corn plants serve as the primary survival strategy for *D. maidis* during the off‐season in Brazil. Research indicates that after the corn season, *D. maidis* populations disperse to these plants and remain until the next planting season.^5^, ^8^, ^10^

The primary strategy to control *D. maidis* has been chemical insecticides, applied either through seed treatment or foliar spraying. Among foliar insecticides used against the corn leafhopper, those containing pyrethroids are the most prominent. Pyrethroid insecticides are often combined with other active ingredients, primarily neonicotinoids. Out of the 88 insecticides registered for use against *D. maidis* in Brazil, 29 contain pyrethroids.^14^ Additionally, pyrethroids are recommended for controlling other sucking insects in corn crops,^14^ such as *Diceraeus melacanthus* (Dallas), *D. furcatus* (Fabricius) (Hemiptera: Pentatomidae), and *Rhopalosiphum maidis* (Fitch) (Hemiptera: Aphididae). Consequently, the use of pyrethroids against other corn pests has also exerted selection pressure on *D. maidis* populations. As a result, the increased selection pressure with pyrethroids may drive resistance evolution, potentially compromising their effectiveness in controlling the corn leafhopper.

Reduced susceptibility to pyrethroids and neonicotinoids due to resistance evolution has been confirmed in Brazil.^6^ Therefore, this study aims to understand *D. maidis* resistance pattern to the pyrethroid bifenthrin to support Insect Resistance Management (IRM) strategies. To achieve this, we selected a bifenthrin‐resistant strain from a field‐collected population of *D. maidis* to characterize the genetic basis of bifenthrin resistance, assess cross‐resistance to other insecticides, and evaluate resistance stability.

## 2 MATERIALS AND METHODS

### 2.1 Insects

The susceptible reference strain (Sus) of *D. maidis* originated from insects collected in corn fields located in the municipality of Jardinópolis (53° 47′ 036″ S; 20° 54′ 46″ W), São Paulo, Brazil. This strain has been maintained under laboratory conditions without exposure to insecticides for over 10 years. The bifenthrin‐resistant strain of *D. maidis* (Bif‐R) was selected under laboratory conditions from a field‐collected population (*n* = 500 insects) in Bt corn fields in Rio Verde, Goiás, Brazil (50° 46′ 57″ S; 17° 06′ 05″ W) in August 2022. *D. maidis* identification was confirmed by examining the genitalia of males and the seventh abdominal sternite of females.^15^

To maintain *D. maidis* strains under controlled laboratory cage conditions, corn plants (hybrid 30F53VYH) with Leptra Technology (Pioneer®, Corteva Agriscience, São Paulo, Brazil) were used at the V_4_–V_5_ development stages. These plants were grown in 415 mL pots (10 cm in diameter, 8 cm in height) containing commercial substrate (Tropstrato HT, Vida Verde Ltda, Mogi‐Mirim, SP) in a greenhouse. The plants were placed inside plastic cages with voile (50 cm height × 35 cm length × 35 cm width) to provide food for the insects. Approximately 500 adults were used for mating and maintaining population density. After 72 h of oviposition, these plants were transferred to cages containing fresh corn plants. Nymphs were reared on fresh plants replaced every 5 days. The insects were kept in a climate‐controlled room at 24 ± 2 °C, relative humidity of 70 ± 10%, and a 14h photoperiod.

### 2.2 Bioassays

Toxicological bioassays were conducted using a modified version of IRAC Method 019 (residual contact method).^6^ Fully expanded leaves from the upper portion of corn plants (hybrid 30F53VYH) were used at the V_6_–V_8_ developmental stages. Leaf discs with a diameter of 3.5 cm were excised using a metal punch. Logarithmically spaced dilutions (32–3,200 μg/mL^−1^) of bifenthrin (Talstar® 100 g a.i./L, FMC Química do Brasil Ltda, Campinas, SP, Brazil) were prepared in distilled water containing 0.1% surfactant (Triton®). The leaf discs were then immersed in these dilutions and allowed to air‐dry. For the control treatment, leaf discs were immersed only in distilled water with 0.1% surfactant.

After drying, the leaf discs were placed on a 3% agar‐water solution in Petri dishes (35 mm × 10 mm). Five‐ to ten‐day‐old adult insects were anesthetized with CO_2_ before being placed on each leaf disc within the Petri dishes. The Petri dishes were then incubated in a BOD chamber at 24 ± 2 °C, with a relative humidity of 70 ± 10% and a 14h photoperiod. Insect mortality was recorded 48 h after exposure.^6^ Insects that showed no movement of wings and legs after gentle prodding with a fine‐bristle brush were considered dead.

### 2.3 Selection of *D. maidis* resistant to bifenthrin

To select the bifenthrin‐resistant strain of *D. maidis* (Bif‐R), the population collected in Rio Verde, Goiás, was divided into two subpopulations. One subpopulation was subjected to selection pressure with bifenthrin resistance for 11 generations (Bif‐R – ‘Selected’), while another subpopulation was maintained in the laboratory without selection pressure for 11 generations (Unselected) in order to observe the increase in resistance frequency in the selected line and verify the decline in the unselected line. The Bif‐R strain was selected using a modified version of IRAC method 05 bioassay.^16^ For this process, corn hybrid 30F53VYH with Leptra Technology (Pioneer®, Corteva Agriscience, São Paulo, Brazil) was sown in 210 mL plastic pots. When the plants reached the V_1_–V_2_ developmental stage, a 3% agar‐water solution was added to the soil surface to prevent the insecticide contact with the substrate. After the agar‐water solution dried, approximately 20 seedlings were submerged for 3 s in an insecticide solution containing bifenthrin and 0.1% surfactant (Triton®), then air‐dried in a flow chamber. The dried seedlings were transferred to rearing cages (50 cm height × 35 cm length × 35 cm width) containing 4th to 5th instar *D. maidis*. After 48 h, the seedlings were removed, and fresh insecticide‐free V_4_–V_5_ stage plants were provided for oviposition. Surviving adult insects initiated the next generation. Artificial selection of resistant insects involved a gradual increase in bifenthrin concentration from 32 to 3,200 μg bifenthrin mL^−1^ (Table S1).

### 2.4 Characterization of *Dalbulus maidis* resistance to bifenthrin

Resistance characterization was conducted on a bifenthrin‐resistant strain of *D. maidis* after nine cycles of selection, as described in Section 2.3. Adult insects from the susceptible strain (5–10 days post‐emergence) were exposed to seven log‐spaced concentrations of bifenthrin ranging from 0.18 to 5.6 μg a.i./mL to achieve 5–95% mortality. Adult insects from the Bif‐R strain were exposed to six concentrations ranging from 320 to 10,000 μg a.i./mL. For the control treatment, only water and surfactant were used.

The experiment followed a completely randomized design, with five replicates per insecticide concentration. Each replicate consisted of eight insects, totaling 40 insects per concentration. Concentration‐mortality data were analyzed using a generalized linear model (GLM) with a binomial distribution and a Probit link function to estimate the LC_50_ values, 95% confidence intervals, and the slopes of the log concentration‐mortality regression lines. LC_50_ values were estimated using the ‘dose.p’ function from MASS package^17^ in R 4.2.1 Software.^18^ The resistance ratio (RR) was calculated by dividing the LC_50_ value of the Bif‐R strain by that of the Sus strain. Tests for parallelism and equality were performed to compare the linear and angular coefficients of the regression lines.^19^

### 2.5 Inheritance pattern of bifenthrin resistance in *D. maidis*

#### 2.5.1 Dominance of resistance

Reciprocal crosses were conducted between resistant (Bif‐R) and susceptible (Sus) adults to produce two strains of heterozygous insects (H1: Bif‐R ♀ × Sus ♂ and H2: Bif‐R ♂ × Sus ♀). For these crosses, glass test tubes (2.4 cm × 10 cm) containing a 3% agar‐water solution and a piece of V_6_‐V_8_ corn leaves were used. Each tube was inoculated with a 5^th^ instar nymph. Upon adult emergence, insects were sexed, with females identified by the presence of ovipositor. Approximately 50 pairs were then placed into rearing cages for the reciprocal crosses. Adults from the resistant, susceptible, and heterozygous (F_1_ generation) strains of *D. maidis* were subjected to bioassays to characterize concentration‐response curves and estimate the LC_50_. The bioassays were conducted as described previously (see Section 2.2).

The estimate of the dominance level of resistance (D) was based on the equation proposed by Bourguet, Genissel, and Raymond^20^:

$$D=\frac{M_{\mathrm{RS}}-M_{\mathrm{SS}}\;}{M_{\mathrm{RR}}-M_{\mathrm{SS}}}$$

In this equation, MRR, MRS, and MSS represent the mortalities at the tested concentrations for the resistant, heterozygous, and susceptible strains, respectively. The value of *D* = 1 suggests complete dominance of the resistance trait, while *D* = 0 indicates complete recessive. The degree of dominance was also assessed using the method proposed by Stone^21^:

$$D=\frac{2\mathrm{XF}-\mathrm{XR}-\mathrm{XS}}{\mathrm{XR}-\mathrm{XS}}$$

where XF, XR, and XS represent the log10 of the LC_50_ values for the heterozygous, resistant, and susceptible insects, respectively. Thus, *D* = 1 indicates complete dominance of resistance, 0 < *D* < 1 suggests incomplete dominance, −1 < *D* < 0 indicates incomplete recessive, and *D* = −1 corresponds to complete recessive.

To assess the functional dominance of resistance in *D. maidis* to bifenthrin, four strains were used: susceptible (Sus), bifenthrin‐resistant (Bif‐R), and heterozygous H1 (♀ Bif‐R × ♂ Sus) and H2 (♀ Sus × ♂ Bif‐R). These strains were tested on corn plants sprayed with bifenthrin at concentrations of 94 and 469 μg a.i mL^−1^, representing low and high recommended field rates for *D. maidis* control. A modified Potter Tower (Burkard Manufacturing, Rickmansworth Herts, UK) calibrated to 10 psi (68.95 kPa) was used to simulate a spraying application according to label recommendations. A volume of 1.5 mL of solution (insecticide + water + surfactant (0.1%)) was applied achieving a spray volume of approximately 160 L per hectare (1.59 mg a.i/cm^2^). The control treatment consisted of spraying only water and surfactant (Triton 0.1%). Each replicate consisted of 10 insects, resulting in a total of 50 insects tested at each treatment. Mortality was evaluated after 48 h.

To compare the mortality rates of different strains at two label concentrations (94 and 469 μg a.i. mL^−1^), a generalized linear model (GLM) with binomial errors was fitted. Model fit was assessed using an analysis of deviance, and verification was performed using a half‐normal plot (hnp) envelope simulation.^22^ Mean mortality rates for each strain were compared using Tukey's test (*p* < 0.05) using ‘emmeans’^23^ and ‘multicomp’^24^ packages. All analyses were conducted using R software version 4.2.1.^18^

#### 2.5.2 Number of genes

To test the hypothesis of monogenic inheritance of resistance, backcrosses were performed between heterozygous insects and the phenotypically distinct homozygous genotype (Sus). Adult offspring from these backcrosses (F_1_) were evaluated in concentration‐response bioassays (see Section 2.2). The potential for monogenic inheritance was analyzed using the chi‐square goodness‐of‐fit test.^25^

$$\chi^{2}=\frac{{\left(\mathrm{Ni}-\text{pqni}\right)}^{2}\;}{\text{pqni}}$$

Where Ni is the observed mortality at concentration i, ni is the number of individuals tested, *q* = 1− *p* and *p* is the expected mortality.^26^

$$p=\frac{a+b\;}{2}$$

The hypothesis of monogenic inheritance was rejected when the calculated *χ* ^2^ value was greater than or equal to the tabulated *χ* ^2^ value.

### 2.6 Cross‐resistance between bifenthrin and other insecticides in *D. maidis*

To investigate the presence of cross‐resistance or multiple resistance of the bifenthrin‐resistant strain (Bif‐R) to other insecticides, concentration‐response curves were determined using the Sus and Bif‐R strains. The insecticides tested included lambda‐cyhalothrin (pyrethroid), imidacloprid, acetamiprid, dinotefuran, and thiamethoxam (neonicotinoid), methomyl and carbosulfan (carbamate), and acephate (organophosphate), as detailed in Table 1. The bioassay method followed the procedures described in Section 2.2. Insect mortality was assessed between 48 and 72 h, depending on the chemical group under analysis.^6^ LC_50_ values for each strain and insecticide were determined as described in Section 2.4. Resistance ratios were calculated by comparing the LC_50_ values of Bif‐R strain to those of the susceptible strain (Sus) for each insecticide to assess potential cross‐resistance.

**Table 1:** Commercial insecticides used to evaluate the cross‐resistance of bifenthrin‐resistant strain of *Dalbulus maidis*

Active ingredient (A.I.)	Insecticide class (IRAC MoA)	Trade name	Manufacturer/company	Range of concentrations* μg a.i./mL
Imidacloprid	Neonicotinoids (4A)	Provado® 200 SC	Bayer S.A., São Paulo, SP, Brazil.	10–1,000
Acetamiprid	Neonicotinoids (4A)	Mospilan WG	Iharabras S.A. Indústrias Químicas, Sorocaba, SP, Brasil.	10–1,000
Dinotefuran	Neonicotinoids (4A)	Dinno	Iharabras S.A. Indústrias Químicas, Sorocaba, SP, Brasil.	3.2–320
Thiamethoxam	Neonicotinoids (4A)	Vivantha	Ouro Fino Química Ltda	3.2–560
Lambda‐cyhalothrin	Pyrethroids (3B)	Kaiso 250 CS	Sumitomo Chemical Brasil Indústria Química S.A	100–10,000
Methomyl	Carbamates (1A)	Lannate® BR	Corteva Agriscience do Brasil S.A., Barueri, SP, Brazil	10–560
Carbosulfan	Carbamates (1A)	Marshal Star™	FMC Química do Brasil Ltda, Campinas, SP, Brazil	1–100
Acephate	Organophosphates (1B)	Perito 970 SG	UPL do Brasil Indústria e Comércio de Insumos Agropecuários S.A., Ituverava, SP, Brazil	1–100

* Range of concentrations used in concentration‐mortality curves.

### 2.7 Stability of bifenthrin resistance in *D. maidis*

To assess the resistance stability in *D. maidis* to bifenthrin, populations with varying proportions of susceptible (S) and bifenthrin‐resistant (R) insects were maintained for six generations in the absence of selection pressure. Five treatments were assessed: (1) 100% resistant insects (100R:0S), (2) 80% resistant and 20% susceptible insects (80R:20S), (3) 50% resistant and 50% susceptible insects (50R:50S), (4) 20% resistant and 80% susceptible insects (20R:80S), and (5) 100% susceptible insects (0R:100S).

To ensure that adults from treatments with different proportions had not mated before, glass test tubes containing a 3% agar‐water solution and V_6_–V_8_ corn leaves were used. Each tube was inoculated with a 5th instar nymph. After emergence, adults were sexed by identifying females based on the visible ovipositor. Subsequently, 10 pairs were formed for each replicate (three) of each treatment (five) and were placed in rearing cages (40 cm height × 35 cm length × 35 cm width). The insects were maintained in a controlled environment at 24 ± 2 °C, 70 ± 10% relative humidity, and a 14h photoperiod. Populations from the five treatments were maintained for six generations on V_4_–V_5_ corn plants within the rearing cages (see Section 2.1).

Throughout six generations, insect susceptibility was assessed using the residual contact bioassay (Section 2.2) with a previously estimated diagnostic concentration of (100 μg bifenthrin mL^−1^) with 0.1% surfactant (Triton®). This concentration was selected based on values close to the upper limit of LC_99_ values of the SUS strain. The experimental design was completely randomized, with 60 replicates per treatment. Each replicate consisted of eight adult insects (aged 5–10 days post‐emergence) in a Petri dish. Mortality was recorded 48 h after exposure to bifenthrin. Insects that showed no apparent movement of wings or legs when gently touched with a fine brush were considered dead.

Resistance stability was assessed using GLM models with a binomial distribution, fitted with the ‘loess’ method. Model fit was evaluated using half‐normal plot (hnp) envelope simulations.^22^ Mean mortality rates for each proportion were compared within each evaluated generation using Tukey's test (*p* < 0.05) with the ‘emmeans’^23^ and ‘multicomp’^24^ packages. All analyses were performed in R version 4.2.1.^18^

## 3 RESULTS

### 3.1 Selection of bifenthrin‐resistant strain of *D. maidis*

**Figure 1:**
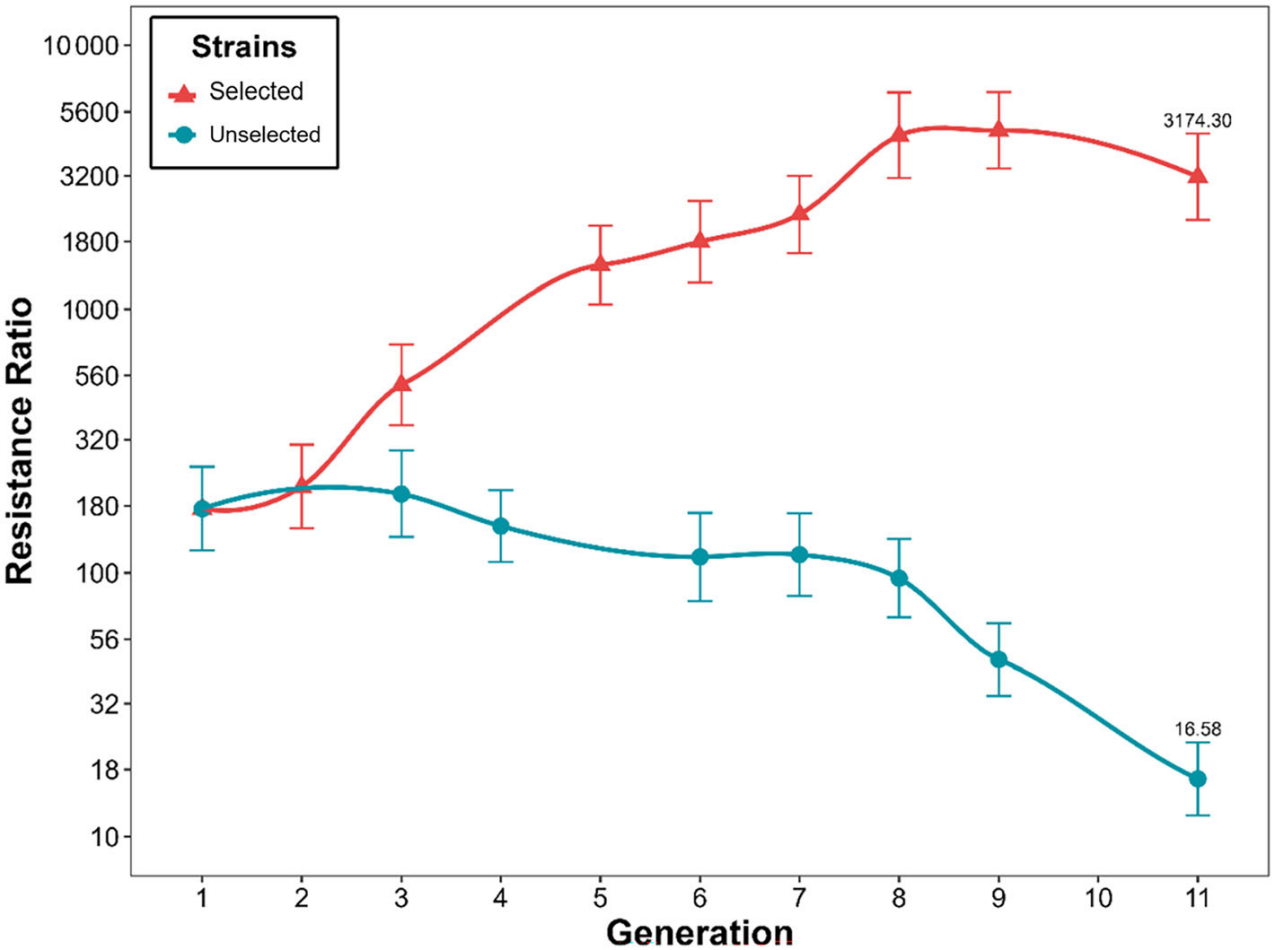
Resistance ratio monitoring during 11 generations in a population of *Dalbulus maidis* in the presence or absence of selection pressure with the insecticide bifenthrin.

LC_50_ value of the field‐collected *D. maidis* population after one laboratory generation (F_1_) was 113.61 (76.51–168.70) μg a.i. mL^−1^, whereas for the Sus strain, it was 0.64 (0.51–0.81) μg a.i. mL^−1^ (Table S2). These values indicate that the field population of *D. maidis* presented a resistance ratio to bifenthrin of 175‐fold. After 11 cycles of selection pressure with concentrations of bifenthrin ranged from 32 to 3,200 μg a.i. mL^−1^, the LC_50_ value of the Bif‐R strain was 2,055.72 (1,297.57–3,256.85) μg a.i. mL^−1^, resulting in a RR > 3,170‐fold. Conversely, the population not subjected to selection pressure increased susceptibility to bifenthrin. The estimated LC_50_ value (95% CI) of the Unselected strain decreased from 113.61 (76.51–168.70) μg bifenthrin mL^−1^ in the first generation (F1) to 10.73 (7.32–15.74) μg bifenthrin mL^−1^ in generation F11, resulting in a RR of only 16‐fold (Fig. 1; Tables S1 and S2).

### 3.2 Inheritance pattern in *D. maidis* resistant to bifenthrin

#### 3.2.1 Dominance of resistance

**Figure 2:**
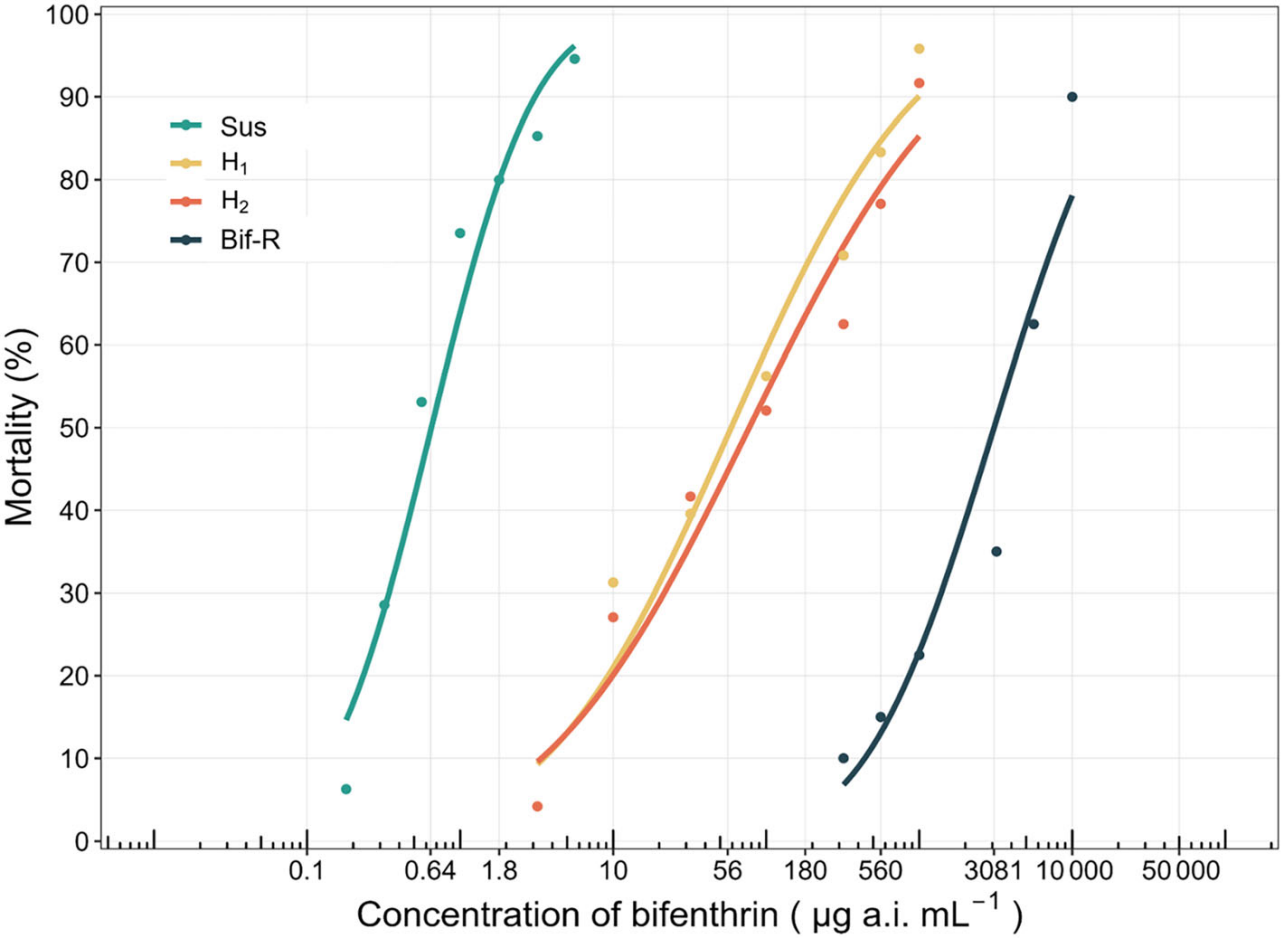
Concentration‐response curves for different *Dalbulus maidis* strains: susceptible (Sus), resistant (Bif‐R), and heterozygous H1 (♀ Bif‐R × ♂ Sus) and H2 (♀ Sus × ♂ Bif‐R) submitted to bifenthrin.

**Figure 3:**
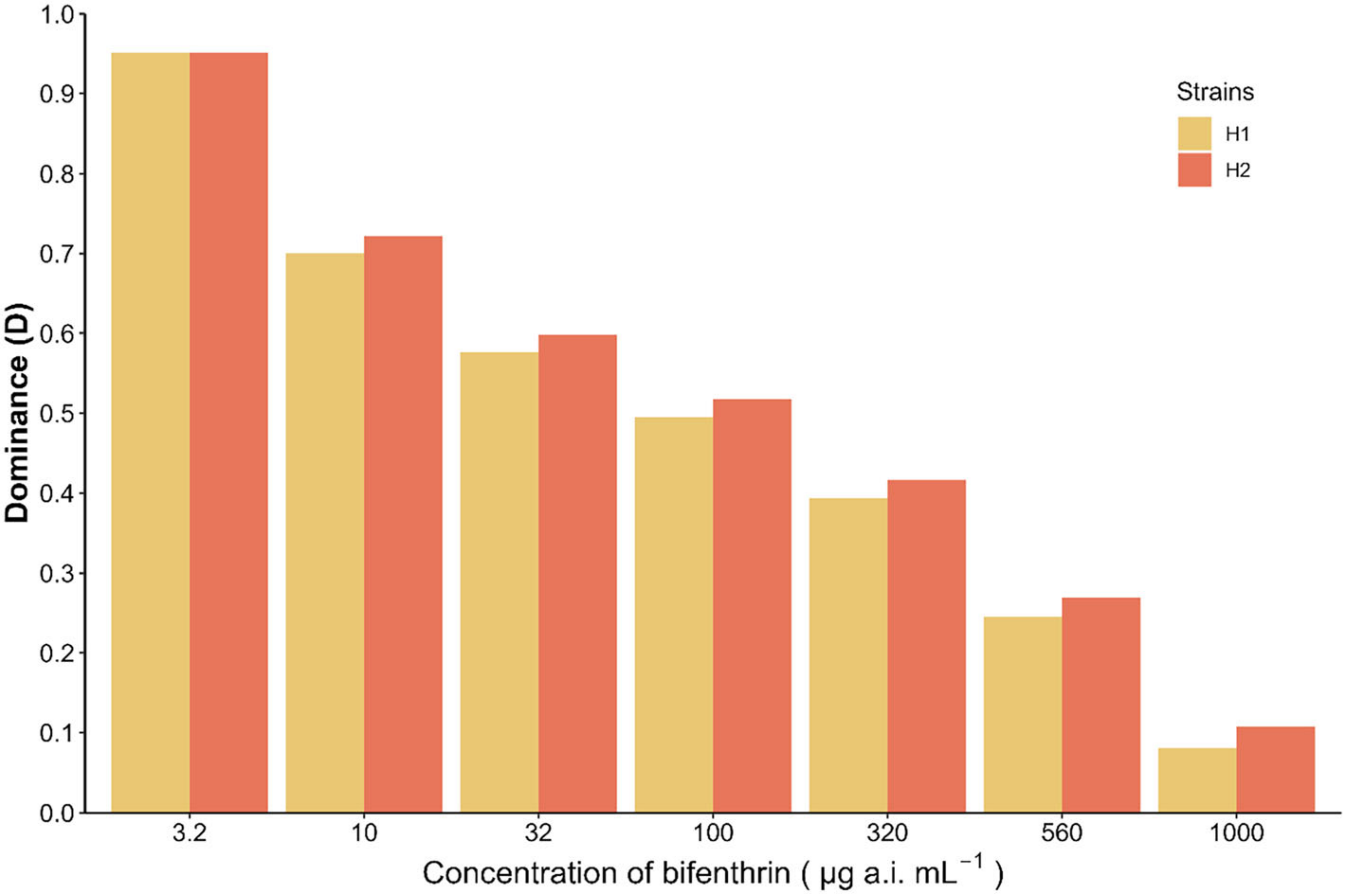
Dominance of *Dalbulus maidis* resistance to bifenthrin in the Bourguet, Genissel, Raymond method. Heterozygous represented by H1 (♀ Bif‐R × ♂ Sus) and H2 (♀ Sus × ♂ Bif‐R).

Concentration‐response bioassays for reciprocal crosses H1 (♀ Bif‐R × ♂ Sus) and H2 (♀ Sus × ♂ Bif‐R) showed no significant differences in tests for equality (*χ* ^2^ = 1.26; degrees of freedom = 2; *p* = 0.533) and parallelism (*χ* ^2^ = 0.47; degrees of freedom = 1; *p* = 0.494), suggesting that the genes associated with resistance in *D. maidis* are located on autosomal chromosomes, and are not maternally inherited or sex‐linked (Table 2, Fig. 2). It was also observed that the dominance level, calculated according to Stone^21^ indicated the resistance is an incomplete dominant trait (H1: *D* = 0.10; H2: *D* = 0.13). According to the Bourguet *et al*.,^20^ the dominance level (DML) calculated decreased as the bifenthrin concentrations increased (Fig. 3).

**Table 2:** Concentration‐mortality response (LC_50_ ± 95% CI) to bifenthrin in susceptible (SUS), resistant (BIF‐R) and heterozygotes H1 (♀ Bif‐R × ♂ Sus) and H2 (♀ Sus × ♂ Bif‐R) strains

Strains	*n* *	Slope ± SE^†^	LC_50_ (95% CI)^‡^ (μg a.i. mL^−1^)	*χ* ^2^ (d.f.)^§^	*P* **	RR^††^
Sus	272	1.88 ± 0.21	0.64 (0.51–0.81)	5,26 (5)	0.15	‐
Bif‐R	280	1.51 ± 0.18	3,080.95 (2,319.32 – 4,092.68)	8.33 (4)	0.08	4,757.53 (3,426.84 – 6,604.96)
H1	336	1.04 ± 0.10	58.98 (41.83–83.17)	7.97 (5)	0.15	91.08 (67.71–122.51)
H2	336	0.94 ± 0.09	77.38 (53.61–111.71)	7.68 (5)	0.17	119.50 (88.41–161.51)

* Number of insects tested;

^†^ Standard error;

^‡^ Lethal concentration 50% and confidence interval (CI) at 95%;

^§^ Degrees of freedom;

** *p* value.

^††^ Resistance ratio: LC_50_ of the resistant strain/LC_50_ of the susceptible strain and 95% confidence interval.

**Figure 4:**
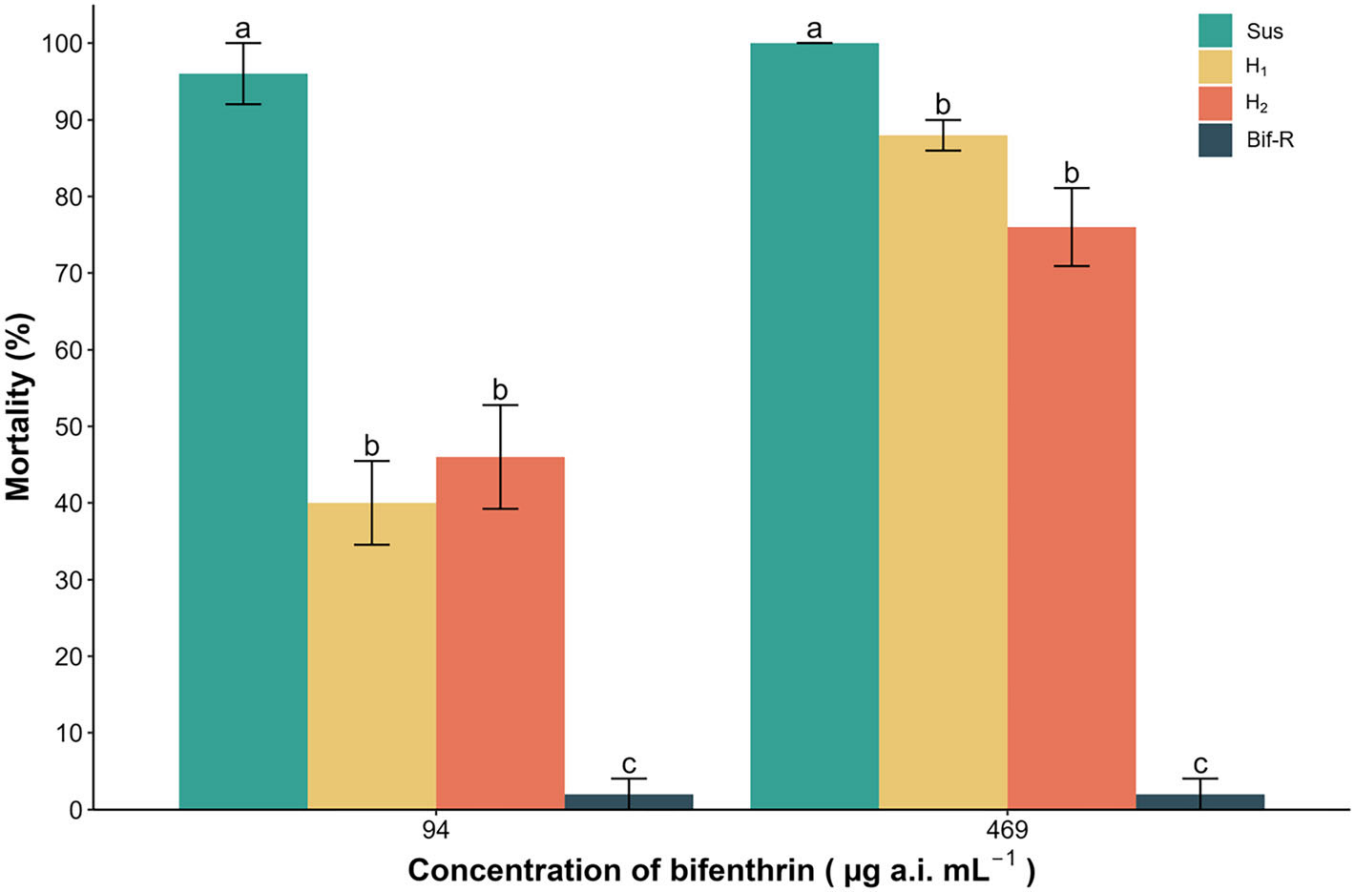
Functional dominance of susceptible, resistant and heterozygous strains (H1 = ♀ Bif‐R × ♂ Sus and H2 = ♀ Sus × ♂ Bif‐R) of *Dalbulus maidis* at low and high concentrations of bifenthrin registered to use in the field. Lowercase letters in bars mean differences between strains at the same concentration.

When evaluating the functional dominance of resistance in *D. maidis* to bifenthrin, statistical differences were observed at both low (94 μg bifenthrin mL^−1^) (*χ* ^2^ = 113.64, *df* = 3, *p* < 0.001) and high concentrations (469 μg bifenthrin mL^−1^) (*χ* ^2^ = 153.46, *df* = 3, *p* < 0.001) field‐recommended concentrations (Fig. 4). At the low concentration, low mortality (<5%) was observed in the Bif‐R, whereas high mortality was observed for the Sus strain (>95%). Both heterozygous strains exhibited similar mortality (<50%), indicating an incomplete dominance inheritance pattern, with dominance levels of 0.60 and 0.53 for H1 and H2 reciprocal cross progeny, respectively. In contrast, at the high concentration, the heterozygous strains exhibited an incomplete recessive inheritance pattern, with dominance levels ranging from 0.12 for H1 and 0.24 for H2. Meanwhile, mortality in the Bif‐R strain remained below 5%, whereas the Sus strain exhibited complete mortality (~100%).

#### 3.2.2 Number of genes associated with resistance

Adults of *D. maidis* from backcrosses exposed to different concentrations of bifenthrin showed a significant deviation between observed and expected mortality (Table 3). The results indicated that the hypothesis of monogenic inheritance was rejected, suggesting that resistance to bifenthrin in *D. maidis* follows a polygenic inheritance pattern, as supported by the calculated *χ* ^2^ values (*p* > 0.05).

**Table 3:** Chi‐square analysis (*χ* ^2^) of mortality from the backcrosses between the susceptible strain (Sus) and the F1 progeny of the reciprocal crosses ((H1 = ♀ Bif‐R × ♂ Sus) and (H2 = ♀ Sus × ♂ Bif‐R)) exposed to different concentrations of bifenthrin

Concentration	♀ Sus × ♂ H1				♂ Sus × ♀ H1				♀ Sus × ♂ H2				♂ Sus × ♀ H2			
μg mL^−1^	Obs*	Exp^†^	*χ* ^2^	*p*	Obs*	Exp^†^	*χ* ^2^	*p*	Obs*	Exp^†^	*χ* ^2^	*p*	Obs*	Exp^†^	*χ* ^2^	*p*
3.2	12.5	45	16.81^‡^	0.00	20.0	45	9.90^‡^	0.00	10.0	45	19.52^‡^	0.00	10.0	45	19.52^‡^	0.00
10	42.5	64	8.26^‡^	0.00	35.0	64	14.93^‡^	0.00	32.5	62	15.00^‡^	0.00	27.5	62	20.47^‡^	0.00
32	65.0	70	0.44	0.51	55.0	70	4.15^‡^	0.04	50.0	71	8.40^‡^	0.00	47.5	71	10.54^‡^	0.00
100	80.0	78	0.08	0.77	75.0	78	0.23	0.63	62.5	76	4.03^‡^	0.04	70.0	76	0.80	0.37
320	87.5	85	0.14	0.70	90.0	85	0.67	0.41	77.5	81	0.37	0.54	82.5	81	0.04	0.83
560	97.5	92	1.78	0.18	97.5	92	1.78	0.18	92.5	89	0.62	0.43	92.5	89	0.62	0.43

* Observed mortality;

^†^ Expected mortality based on Mendelian inheritance;

^‡^ Significant difference (*p* < 0.05, degree of freedom = 1) between observed and expected mortality.

### 3.3 Cross‐resistance between bifenthrin and other insecticides in *D. maidis*

The Bif‐R strain exposed to insecticides with distinct modes of action, including carbamates (methomyl and carbosulfan), organophosphates (acephate), and neonicotinoids (dinotefuran and thiamethoxam) exhibited no cross‐resistance (RR < 10‐fold). Conversely, possible cross‐resistance was observed when adults of *D. maidis* were exposed to the pyrethroid lambda‐cyhalothrin (RR ≈ 2,000‐fold), along with potential multiple resistance to the neonicotinoids acetamiprid and imidacloprid (RR ranging from 300 to >600‐fold) (Table 4).

**Table 4:** Concentration‐mortality response (LC_50_ ± 95% CI) of resistant and susceptible *Dalbulus maidis* strains exposed to different insecticides

Insecticides	*n* *	Slope ± SE^†^	*χ* ^2^ (d.f.)^‡^	*p* ^§^	LC_50_ (95% CI)** (μg i.a. mL^−1^)	RR^††^
Nicotinic acetylcholine receptor (nAChR) competitive modulators (Irac Group 4A)						
Imidacloprid						
Bif‐R	240	1.05 ± 0.13	6.34 (4)	0.17	74.62 (49.81–111.79)	621.83
Sus	320	1.23 ± 0.12	3.50 (6)	0.74	0.12 (0.08–0.17)	‐
Acetamiprid						
Bif‐R	240	1.21 ± 0.14	8.03 (4)	0.09	132.26 (93.94–186.21)	314.90
Sus	320	1.14 ± 0.11	3.76 (6)	0.70	0.42 (0.32–0.54)	‐
Dinotefuran						
Bif‐R	240	1.10 ± 0.15	5.89 (3)	0.11	33.17 (22.21–49.55)	4.01
Sus	240	1.32 ± 0.16	1.62 (3)	0.65	8.26 (5.82–11.73)	‐
Thiamethoxam						
Bif‐R	240	1.10 ± 0.12	6.20 (4)	0.18	68.13 (46.66–99.49)	6.00
Sus	280	1.02 ± 0.12	5.86 (4)	0.20	11.34 (7.55–17.02)	‐
Sodium channel modulators (Irac Group 3A)						
Lambda‐cyhalothrin						
Bif‐R	280	1.14 ± 0.13	4.27 (5)	0.51	1,431.76 (1,032.49 – 1,985.42)	2,045.37
Sus	280	1.16 ± 0.15	2.75 (4)	0.59	0.70 (0.49–0.99)	‐
Acetylcholinesterase (AChE) inhibitors (Irac Group 1A)						
Methomyl						
Bif‐R	280	1.21 ± 0.11	7.15 (6)	0.30	45.58 (33.34–62.31)	3.56
Sus	280	2.29 ± 0.27	2.88 (4)	0.57	12.79 (10.04–14.56)	‐
Carbosulfan						
Bif‐R	240	1.49 ± 0.15	3.66 (6)	0.72	60.07 (47.22–76.42)	2.94
Sus	280	1.62 ± 0.18	3.06 (4)	0.54	20.40 (15.62–26.23)	‐
Acetylcholinesterase (AChE) inhibitors (Irac Group 1B)						
Acephate						
Bif‐R	240	1.55 ± 0.17	3.25 (4)	0.51	9.63 (7.29–12.73)	1.52
Sus	280	2.46 ± 0.29	0.98 (4)	0.91	6.33 (5.24–7.75)	‐

* Number of insects tested.

^†^ Standard error.

^‡^ Lethal concentration 50% and confidence interval (CI) at 95%.

^§^ Degrees of freedom.

** *p* value.

^††^ Resistance ratio LC_50_ of the resistant lineage/ LC_50_ of the susceptible lineage.

### 3.4 Stability of *D. maidis* resistance to bifenthrin

**Figure 5:**
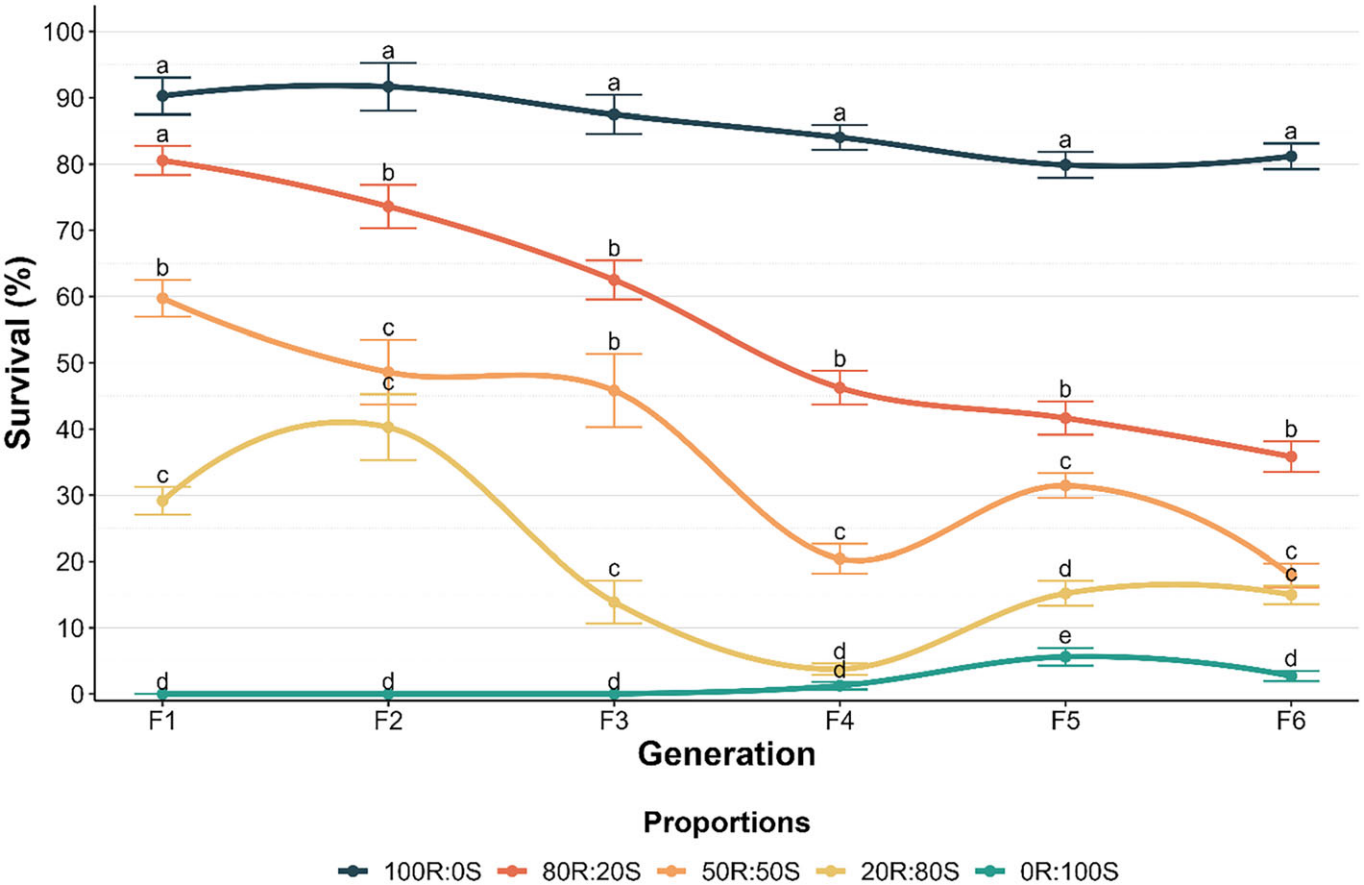
Survival (%) at a diagnostic concentration (100 μg a.i. mL^−1^) in *Dalbulus maidis* populations with different proportions of resistant and susceptible insects. Lowercase letters in bars mean differences between strains at the same generation.

Over six generations in the absence of selection pressure with bifenthrin, increased susceptibility was observed in treatments with different proportions of bifenthrin‐resistant (R) and susceptible (S) individuals (Fig. 5). The LC_50_ values for the 0R:100S and 20R:80S proportions remained stable, ranging from 0.75 to 0.72 μg bifenthrin mL^−1^, and 17 to 13 μg bifenthrin mL^−1^, respectively (Table S3). In contrast, the LC_50_ values for the 100R:0S, 80R:20S, and 50R:50S proportions decreased from 2,900 to 1,500, 930 to 69 and 150 to 18 μg bifenthrin mL^−1^, reducing the resistance ratio to 2,120‐, 95‐, and 25‐fold, respectively (Fig. 5 and Table S3).

## 4 DISCUSSION

In this study, we characterized the resistance of *D. maidis* to bifenthrin. Our findings explain the rapid evolution of resistance to this insecticide and provide insight into the reduced susceptibility to bifenthrin in different regions in Brazil.^6^ The field‐collected population exhibited a resistance ratio of approximately 175‐fold in the first laboratory generation, highlighting a high frequency of resistant *D. maidis* under field conditions.

The inheritance pattern of the bifenthrin‐resistant strain of *Dalbulus maidis* (Bif‐R) was autosomal and incompletely dominant. These findings align with previous studies on other sucking pests. For example, *Amrasca biguttula biguttula* (Hemiptera: Cicadellidae) exhibited an autosomal and incompletely dominant resistance pattern in dimethoate.^27^ Similar resistance patterns have also been observed in *Bemisia tabaci* (Hemiptera: Aleyrodidae) and *Oxycarenus hyalinipennis* (Hemiptera: Lygaeidae), with resistance to bifenthrin characterized as autosomal and incompletely dominant.^28^, ^29^ The dominant resistance inheritance observed in our study supports the evolution of resistance, primarily due to the survival of heterozygotes, which increases the frequency of individuals carrying the resistance allele in the field.^30^ This inheritance pattern likely contributes to field failures associated with pyrethroid use in certain regions of Brazil.

The inheritance pattern was functionally recessive at high bifenthrin concentrations. These findings suggest that dominance is not a fixed genetic parameter but varies with the dose. This variability has important implications for resistance management strategies. At low bifenthrin doses, heterozygous individuals may survive, potentially increasing the frequency of resistant alleles in the field, which could lead to control failures. Conversely, high doses may slow resistance evolution by eliminating heterozygotes. However, the insecticide's biological activity diminishes over time, as does the amount of active ingredient that reaches the target in the field (due to challenges in the application of technology) also contributes to a lower dose of products, allowing some heterozygous individuals to survive. Moreover, caution is needed when applying doses higher than recommended, as excessive use may select for more resistant insects and increase environmental impact.

While pyrethroid resistance mechanisms have been linked to target‐site mutations, such as knockdown resistance (kdr) mutations,^31^, ^32^, ^33^, ^34^, ^35^ evidence also suggests a role for metabolic processes and cuticle thickening.^36^, ^37^, ^38^, ^39^ Given the diversity of genes involved in metabolic pathways and target‐site mutations in insects, multiple *loci* may contribute to bifenthrin resistance in *D. maidis*. Our findings indicate that bifenthrin resistance in *D. maidis* is a polygenic trait. Similar polygenic resistance patterns have been observed in other hopper species, such as *Laodelphax striatellus* (Hemiptera: Delphacidae) resistant to triflumezopyrim^40^ and *Nilaparvata lugens* (Hemiptera: Delphacidae) resistant to imidacloprid.^41^ Since multiple genes are required to confer resistance, the evolution of polygenic resistance in the field tends to be slower. When a polygenically resistant individual disperses into a susceptible population, resistant alleles are more rapidly diluted, reducing the likelihood of resistance expression. In the absence of selection pressure, resistant individuals are not favored, and their frequency may decline if resistance is associated with fitness costs.^30^, ^42^ Further research is needed to elucidate the molecular mechanisms underlying resistance to bifenthrin in *D. maidis*.

The bifenthrin‐resistant strain (Bif‐R) exhibited high levels of potential cross‐resistance to lambda‐cyhalothrin. Additionally, the high resistance ratios observed for imidacloprid and acetamiprid suggest potential multiple resistance to neonicotinoids. Similar results were found in aphid field populations in China, showing broad resistance to neonicotinoids and pyrethroids. This resistance involves mutations (*R81T* and *kdr*), indicating target‐site resistance and various enzymes like monooxygenase, carboxylesterase, superoxide dismutase, and peroxidase, contributing to multiple resistance in these aphids.^43^ Frequent use of neonicotinoids in combination with pyrethroids (e.g., imidacloprid with bifenthrin formulated products) for *D. maidis* control may have selected resistant populations to both insecticides. This could undermine strategies involving the rotation or mixing of insecticides with these chemical groups.^6^

Field populations of *D. maidis* across different regions in Brazil showed reduced susceptibility to certain pyrethroid and neonicotinoid insecticides, suggesting that some active ingredients of these chemical groups may no longer be as effective as they once were.^6^ In this context, the resistance of *D. maidis* to bifenthrin, combined with cross‐resistance to lambda‐cyhalothrin and potential multiple resistance to imidacloprid and acetamiprid, makes the use of these insecticides problematic for managing *D. maidis* resistance in regions with a high frequency of resistant insects. On the other hand, no cross‐resistance was observed for acetylcholinesterase‐inhibiting insecticides such as carbosulfan and methomyl (carbamates–IRAC Group 1A) or acephate (organophosphate–IRAC Group 1A). Similarly, no cross‐resistance was detected for nicotinic acetylcholine receptor (nAChR) competitive modulator insecticides such as thiamethoxam and dinotefuran (neonicotinoids–IRAC MoA 4), the lack of resistance to these neonicotinoids may be related to the molecular structure. The chemical structures of insecticides vary significantly according to the generation, and their interaction with the target site in the insect may differ. These insecticides could be viable alternatives for use in rotation strategies to help manage *D. maidis* resistance to bifenthrin.

Knowledge of resistance stability is crucial for effective resistance management strategies. If resistance is unstable, avoiding the use of that insecticide should reduce the frequency of resistant individuals over time due to the fitness costs associated with resistance in the absence of selection pressure, potentially slowing the evolution of resistance.^44^ Our results showed that the resistance of *D. maidis* to bifenthrin was unstable. When the Bif‐R strain was maintained for six generations without selection pressure from bifenthrin, the resistance ratio decreased from 3,880‐fold to 2,120‐fold (about 2‐fold). In comparison, the unselected strain after 11 generations had a reduction from 113‐fold to 10‐fold. This difference (2‐fold *vs*. 10‐fold) is probably due to the higher frequency of susceptible individuals in the original field population, which is consistent with the mixed population experiment. Previous studies have reported fitness costs associated with resistance to the pyrethroid esfenvalerate in *N. lugens* ^45^ and in other bifenthrin‐resistant sucking insects, such as *B. tabaci* and *O. hyalinipennis*.^28^, ^29^ Incorporating fitness costs into insecticide resistance management (IRM) strategies can help delay the increase in allele frequencies responsible for resistance or even restore susceptibility.^46^, ^47^, ^48^

Strategies to manage resistance by utilizing fitness costs can include increasing temporal refuges through insecticide rotation and using host plants or varieties that show a greater magnitude of fitness cost.^47^, ^49^, ^50^, ^51^ The goal is to alter the frequency of resistant individuals in the population, allowing susceptible phenotypes a greater chance of survival.^47^, ^52^ In this study, we demonstrated that after six generations, when susceptible individuals were introduced into the system under laboratory conditions, a reduction in the resistance ratios of up to 13‐fold was observed. This reduction was reflected in a 45% decrease in survival rate, even when the initial population consisted of 80% of resistant individuals.

Our findings indicate that this resistance is unstable, a characteristic that should be exploited in resistance management programs. Effective strategies include implementing fallow periods, eliminating volunteer corn before replanting, rotating crops, and conserving natural predators and parasitoids within the agricultural landscape. Additionally, sustainable practices such as applying entomopathogenic fungi and cultivating plant varieties that are more tolerant or resistant to *D. maidis* can contribute to pest management.^4^, ^9^, ^53^, ^54^, ^55^, ^56^ The use of active ingredients from different chemical groups in an insecticide rotation system—such as carbamates and organophosphates—and selecting insecticides that do not exhibit cross‐ or multiple‐resistance is crucial for reducing selection pressure on *D. maidis* population with bifenthrin.

This study is the first comprehensive analysis of pyrethroid resistance in *D. maidis* and will contribute to insect resistance management (IRM) strategies to preserve the efficacy of bifenthrin and other insecticides.

## Supporting information


**Table S1.** Concentration‐mortality response (LC_50_ ± 95% CI) to bifenthrin during 11 generations in a population of *Dalbulus maidis* collected in a commercial corn field in Rio Verde, Goiás, Brasil.

**Table S2.** Concentration‐mortality response (LC50 ± 95% CI) in the absence of selection pressure with bifenthrin during 11 generations in a population of *Dalbulus maidis* collected in a commercial corn field in Rio Verde, Goiás, Brazil.

**Table S3.** Concentration‐response of the treatments 100R:0S, 80R:20S, 50R:50S, 20R:80S, and 0R:100S of *Dalbulus maidis* to the insecticide bifenthrin.

## Data Availability

The data that support the findings of this study are available from the corresponding author upon reasonable request.

## References

[1] Nault LR and Delong DM , Evidence for co‐evolution of leafhoppers in the genus *Dalbulus* (Cicadellidae: Homoptera) with maize and its ancestors. Ann Entomol Soc Am 73:349–353 (1980). 10.1093/aesa/73.4.349.

[2] Nault LR , Evolution of an insect pest: maize and the corn leafhopper, a case study. Maydica 35:165–175 (1990). 10.5555/19901150814.

[3] Vilanova ES , Ramos A , de Oliveira MCS , Esteves MB , Gonçalves MC and Lopes JRS , First report of a Mastrevirus (*Geminiviridae*) transmitted by the corn leafhopper. Plant Dis 106:1330–1333 (2022). 10.1094/PDIS-09-21-1882-SC. 34854758

[4] de Oliveira CM and Frizzas MR , Eight decades of *Dalbulus maidis* (DeLong & Wolcott) (Hemiptera, Cicadellidae) in Brazil: what we know and what we need to know. Neotrop Entomol 51:1–17 (2022). 10.1007/s13744-021-00932-9. 34878633

[5] Oliveira CM , Lopes JRS and Nault LR , Survival strategies of *Dalbulus maidis* during maize off‐season in Brazil. Entomol Exp Appl 147:141–153 (2013). 10.1111/eea.12059.

[6] Machado EP , Souza EV , Dias GS , Sacilotto MG and Omoto C , Is insecticide resistance a factor contributing to the increasing problems with *Dalbulus maidis* (Hemiptera: Cicadellidae) in Brazil? Pest Manag Sci 80:5120–5130 (2024). 10.1002/ps.8237. 38868923

[7] Bellota E , Dávila‐Flores A and Bernal JS , A bird in the hand versus two in the bush? The specialist leafhopper *Dalbulus maidis* (Hemiptera: Cicadellidae) does not discriminate against sub‐optimal host plants (*Zea* spp.). Neotrop Entomol 47:171–180 (2018). 10.1007/s13744-017-0516-0. 28397144

[8] Sabato EO , Karam D and Oliveira CM , Sobrevivência da cigarrinha *Dalbulus maidis* (Hemiptera Cicadellidae) em espécies de plantas da família Poaceae http://www.infoteca.cnptia.embrapa.br/infoteca/handle/doc/1100098 [24 February 2025].

[9] Pozebon H , Stürmer GR and Arnemann JA , Corn stunt Pathosystem and its leafhopper vector in Brazil. J Econ Entomol 115:1817–1833 (2022). 36130194 10.1093/jee/toac147

[10] Oliveira CM , Frizzas MR and de Oliveira E , Overwintering plants for *Dalbulus maidis* (DeLong and Wolcott) (Hemiptera: Cicadellidae) adults during the maize off‐season in central Brazil. Int J Trop Insect Sci 40:1105–1111 (2020). 10.1007/s42690-020-00165-0.

[11] Santana PA , Kumar L , Da Silva RS , Pereira JL and Picanço MC , Assessing the impact of climate change on the worldwide distribution of *Dalbulus maidis* (DeLong) using MaxEnt. Pest Manag Sci 75:2706–2715 (2019). 10.1002/ps.5379. 30779307

[12] Van Nieuwenhove GA , Frías EA and Virla EG , Effects of temperature on the development, performance and fitness of the corn leafhopper *Dalbulus maidis* (DeLong) (Hemiptera: Cicadellidae): implications on its distribution under climate change. Agric For Entomol 18:1–10 (2016). 10.1111/afe.12118.

[13] Coll‐Aráoz MV , Hill JG , Albarracin E , Virla EG and Fernandez PC , Modern maize hybrids have lost volatile bottom‐up and top‐down control of *Dalbulus maidis*, a specialist herbivore. J Chem Ecol 46:906–915 (2020). 10.1007/s10886-020-01204-3. 32715406

[14] Ministério da Agricultura Pecuária e Abastecimento , Sistema de agrotóxicos fitossanitários http://extranet.agricultura.gov.br/agrofit_cons/principal_agrofit_cons [24 February 2025].

[15] Triplehorn BW and Nault LR , Phylogenetic classification of the genus *Dalbulus* (Homoptera: Cicadellidae), and notes on the phylogeny of the Macrostelini. Ann Entomol Soc Am 78:291–315 (1985). 10.1093/aesa/78.3.291.

[16] Insecticide Resistance Action Committee (IRAC) , IRAC susceptibility test method No 005 https://irac-online.org/methods/nilaparvata-lugens-nephotettix-cincticeps-adults/ [11 February 2025].

[17] Ripley B , Venables B , Bates DM , Hornik K , Gebhardt A and Firth D , Package ‘MASS’ (Version 7.3‐51.4). Cran‐R Project https://cran.r-project.org/web/packages/MASS/index.html [18 July 2024].

[18] R Development Core Team , R: A Language and Environment for Statistical Computing. R Foundation for Statistical Computing, Vienna, Austria (2023) https://www.R-project.org/. [10 January 2025].

[19] Robertson JL , Jones MM , Olguin E and Alberts B , Bioassays with Arthropods, 3rd edn. CRC Press, Boca Raton, p. 220 (2017).

[20] Bourguet D , Genissel A and Raymond M , Insecticide resistance and dominance levels. J Econ Entomol 93:1588–1595 (2000). 10.1603/0022-0493-93.6.1588. 11142285

[21] Stone BF , A formula for determining degree of dominance in cases of monofactorial inheritance of resistance to chemicals. Bull World Health Organ 38:325–326 (1968). 5302309 PMC2554319

[22] Moral RA , Hinde J and Demétrio CGB , Half‐Normal plots and overdispersed models in *R*: the hnp package. J Stat Softw 81:1–23 (2017).

[23] Lenth RV , R package version 1.10.2.090002. 2024. emmeans: Estimated Marginal Means, aka Least‐Squares Means https://cran.r-project.org/web/packages/emmeans/emmeans.pdf [19 February 2025].

[24] Hothorn T , Bretz F and Westfall P , Simultaneous inference in general parametric models. Biom J 50:346–363 (2008). 10.1002/bimj.200810425. 18481363

[25] Sokal RR and Rohlf FJ , Biometry: The Principles and Practice of Statistics in Biological Research, 3rd edn. W.H. Freeman and Co, New York (1995).

[26] Georghiou G and Taylor C , Factors influencing the evolution of resistance, in National Academy Press Pesticide Resistance: Strategies and Tactics for Management. National Academy Press, Washington, pp. 157–169 (1986).

[27] Khalid I , Mohsin M , Abubakar M , Ali Shad S and Binyameen M , Polygenic and autosomally inherited dimethoate resistance in *Amrasca biguttula biguttula* with no cross‐resistance to bifenthrin, imidacloprid, and chlorfenapyr. Crop Prot 163:106099 (2023).

[28] Basit M , Status of insecticide resistance in *Bemisia tabaci*: resistance, cross‐resistance, stability of resistance, genetics and fitness costs. Phytoparasitica 47:207–225 (2019). 10.1007/s12600-019-00722-5.

[29] Banazeer A , Usama Khan HM , Shahzad Afzal MB and Shad SA , Characterization of genetic basis and realized heritability of bifenthrin‐resistance selected in dusky cotton bug, *Oxycarenus hyalinipennis* (costa) (Hemiptera: Lygaeidae) in Pakistan. Crop Prot 141:105441 (2021).

[30] Roush RT and McKenzie JA , Ecological genetics of insecticide and acaricide resistance. Annu Rev Entomol 32:361–380 (1987). 10.1146/annurev.en.32.010187.002045. 3545056

[31] Chen M , Du Y , Zhu G , Takamatsu G , Ihara M , Matsuda K *et al*., Action of six pyrethrins purified from the botanical insecticide pyrethrum on cockroach sodium channels expressed in *Xenopus* oocytes. Pestic Biochem Physiol 151:82–89 (2018). 30704718 10.1016/j.pestbp.2018.05.002

[32] Dong K , Insect sodium channels and insecticide resistance. Invert Neurosci 7:17–30 (2007). 10.1007/s10158-006-0036-9. 17206406 PMC3052376

[33] Dong K , Du Y , Rinkevich F , Nomura Y , Xu P , Wang L *et al*., Molecular biology of insect sodium channels and pyrethroid resistance. Insect Biochem Mol Biol 50:1–17 (2014). 24704279 10.1016/j.ibmb.2014.03.012 PMC4484874

[34] Du Y , Nomura Y , Zhorov BS and Dong K , Molecular mechanism of knockdown resistance to pyrethroid insecticides. Acta Hortic 1169:25–32 (2017).

[35] Hu Z , Du Y , Nomura Y and Dong K , A sodium channel mutation identified in Aedes aegypti selectively reduces cockroach sodium channel sensitivity to type I, but not type II pyrethroids. Insect Biochem Mol Biol 41:9–13 (2011). 20869441 10.1016/j.ibmb.2010.09.005 PMC3022105

[36] Balabanidou V , Kampouraki A , MacLean M , Blomquist GJ , Tittiger C , Juárez MP *et al*., Cytochrome P450 associated with insecticide resistance catalyzes cuticular hydrocarbon production in *Anopheles gambiae* . Proc Natl Acad Sci 113:9268–9273 (2016). 10.1073/pnas.1608295113. 27439866 PMC4995928

[37] Lira EC , Nascimento AR , Bass C , Omoto C and Cônsoli FL , Transcriptomic investigation of the molecular mechanisms underlying resistance to the neonicotinoid thiamethoxam and the pyrethroid lambda‐cyhalothrin in *Euschistus heros* (Hemiptera: Pentatomidae). Pest Manag Sci 79:5349–5361 (2023). 10.1002/ps.7745. 37624650

[38] Sosa‐Gómez DR , Corrêa‐Ferreira BS , Kraemer B , Pasini A , Husch PE , Delfino Vieira CE *et al*., Prevalence, damage, management and insecticide resistance of stink bug populations (Hemiptera: Pentatomidae) in commodity crops. Agric For Entomol 22:99–118 (2019). 10.1111/afe.12366.

[39] Wu L , Yu Z , Jia Q , Zhang X , Ma E , Li S *et al*., Knockdown of LmCYP303A1 alters cuticular hydrocarbon profiles and increases the susceptibility to desiccation and insecticides in *Locusta migratoria* . Pestic Biochem Physiol 168:104637 (2020). 32711771 10.1016/j.pestbp.2020.104637

[40] Zhang S , Gu F , Du Y , Li X , Gong C , Pu J *et al*., Risk assessment and resistance inheritance of triflumezopyrim resistance in *Laodelphax striatellus* . Pest Manag Sci 78:2851–2859 (2022). 10.1002/ps.6909. 35393666

[41] Sanada‐Morimura S , Fujii T , Van Chien H , Cuong LQ , Estoy GF and Matsumura M , Selection for imidacloprid resistance and mode of inheritance in the brown planthopper, Nilaparvata lugens. Pest Manag Sci 75:2271–2277 (2019). 10.1002/ps.5364. 30701654

[42] Georghiou GP and Taylor CE , Genetic and biological influences in the evolution of insecticide resistance. J Econ Entomol 70:319–323 (1977). 874142 10.1093/jee/70.3.319

[43] Hu J , Chen F , Wang J , Rao W , Lin L and Fan G , Multiple insecticide resistance and associated metabolic‐based mechanisms in a *Myzus persicae* (Sulzer) population. Agronomy 13:2276 (2023).

[44] Dennehy TJ , Nyrop JP and Martinson TE , Characterization and exploitation of instability of spider mite resistance to acaricides, in Managing resistance to agrochemicals, ed. by Green MB , HM LB and Moberg WK . American Chemical Society, Washington pp. 77–91 (1990).

[45] Ling S , Zhang H and Zhang R , Effect of fenvalerate on the reproduction and fitness costs of the brown planthopper, *Nilaparvata lugens* and its resistance mechanism. Pestic Biochem Physiol 101:148–153 (2011).

[46] Brown DJ and Redak RA , Fitness costs associated with insecticide resistance in populations of *Homalodisca vitripennis* Germar (Hemiptera: Cicadellidae). J Econ Entomol 116:560–564 (2023). 36708025 10.1093/jee/toad009

[47] Gassmann AJ , Fitness costs of resistance and their potential application for insect resistance management, in Insect Resistance Management, ed. by Onstad DW and Knolhoff LM . Elsevier Science Publishers, London, pp. 465–491 (2023).

[48] Qin Y , Xu P , Jin R , Li Z , Ma K , Wan H *et al*., Resistance of *Nilaparvata lugens* (Hemiptera: Delphacidae) to triflumezopyrim: inheritance and fitness costs. Pest Manag Sci 77:5566–5575 (2021). 10.1002/ps.6598. 34390298

[49] Kanno RH , Guidolin AS , Padovez FEO , Rodrigues JG and Omoto C , Fitness costs associated with spinetoram resistance in *Spodoptera frugiperda* is driven by host plants. J Pest Sci 96:1625–1635 (2004). 10.1007/s10340-023-01614-8.

[50] Carrière Y , Yelich AJ , Degain BA , Harpold VS , Unnithan GC , Kim JH *et al*., Gossypol in cottonseed increases the fitness cost of resistance to Bt cotton in pink bollworm. Crop Prot 126:104914 (2019).

[51] Padovez FEO , Kanno RH , Zambon GZ , Omoto C and Guidolin AS , The cost of resistance to diamide insecticide varies with the host plant in *Spodoptera frugiperda* (Lepidoptera: Noctuidae). J Econ Entomol 115:2041–2050 (2022). 36255734 10.1093/jee/toac160

[52] Tabashnik BE and Carrière Y , Surge in insect resistance to transgenic crops and prospects for sustainability. Nat Biotechnol 35:926–935 (2017). 29020006 10.1038/nbt.3974

[53] Moya‐Raygoza G , Renteria CI , Albarracin EL and Virla EG , Egg parasitoids of the leafhoppers *Dalbulus maidis* and *Dalbulus elimatus* (Hemiptera: Cicadellidae) in two maize habitats. Fla Entomol 97:309–312 (2014).

[54] Virla EG , Moya‐Raygoza G and Luft‐Albarracin E , Egg parasitoids of the corn leafhopper, *Dalbulus maidis*, in the southernmost area of its distribution range. J Insect Sci 13:1–7 (2013). 10.1673/031.013.1001. 23879879 PMC3735118

[55] Albarracin EL , Triapitsyn SV and Virla EG , Egg parasitoid complex of the corn leafhopper, *Dalbulus maidis* (DeLong) (Hemiptera: Cicadellidae), in Argentina. Neotrop Entomol 46:666–677 (2017). 28643143 10.1007/s13744-017-0535-x

[56] Maluta N , Fereres A and Lopes JRS , Plant‐mediated indirect effects of two viruses with different transmission modes on *Bemisia tabaci* feeding behavior and fitness. J Pest Sci 92:405–416 (2019).

