# Supplementary material for: Bifenthrin resistance in Dalbulus maidis (Hemiptera: Cicadellidae): inheritance, cross‐resistance, and stability
Source: Pest Manag Sci. 2025 Apr 25;81(8):4810–20. doi: 10.1002/ps.8848 (PMC12268804; doi:10.1002/ps.8848)
Supplement: Supplementary file 1 — Table S1. Concentration‐mortality response (LC50 ± 95% CI) to bifenthrin during 11 generations in a population of Dalbulus maidis collected in a commercial corn field in Rio Verde, Goiás, Brasil. [file PS-81-4810-s002.docx]

**Table S1.** Concentration-mortality response (LC_50_ ± 95%CI) to bifenthrin during eleven generations in a population of *D. maidis* collected in a commercial corn field in Rio Verde, Goiás, Brasil.

| **Strain**  **(generation)** | **n^a^** | **Slope ± SE^b^** | **LC_50_ (95% CI)^c^**  **(μg a.i. ml^-1^)** | **χ² (d.f.)^d^** | ***p*^e^** | **Concentration used for selection (μg a.i. ml^-1^)** | **RR_50_^f^** |
| --- | --- | --- | --- | --- | --- | --- | --- |
| Sus | 272 | 1.88 ± 0.21 | 0.64 (0.51 – 0.81) | 5,26 (5) | 0.38 | - | - |
| **(F_1_)** | 281 | 1.00 ± 0.14 | 113.61 (76.51 – 168.70) | 3.83 (4) | 0.42 | 32 | 175.43 (122.00 – 252.24) |
| **(F_2_)** | 281 | 0.98 ± 0.14 | 137.68 (91.31 – 207.59) | 3.51 (4) | 0.47 | 100 | 212.60 (147.67 – 306.08 |
| **(F_3_)** | 314 | 1.10 ± 0.15 | 334.36 (224.55 – 497.85) | 1.29 (4) | 0.86 | 320 | 516.29 (362.26 – 735.82) |
| **(F_5_)** | 320 | 1.20 ± 0.15 | 951.09 (702.76 – 1,287.18) | 8.08 (5) | 0.15 | 1,000 | 1,486.61 (1,040.55 – 2,072.78) |
| **(F_6_)** | 320 | 1.12 ± 0.11 | 1,165.89 (842.61– 1,613.20) | 4.03 (5) | 0.54 | 1,000 | 1,800.29 (1,260.36 – 2,571.53) |
| **(F_7_)** | 320 | 1.34 ± 0.17 | 1,479.92 (1,112.22 – 1,969.18) | 5.90 (5) | 0.31 | 1,000 | 2,285.19 (1,630.06 – 3,203.62) |
| **(F_8_)** | 280 | 1.16 ± 0.17 | 2,951.99 (1,897.99 – 4,591.30) | 6.01 (4) | 0.19 | 3,200 | 4,558.25 (3,140.12 – 6,616.83) |
| **(F_9_)** | 280 | 1.51 ± 0.18 | 3,080.95 (2,319.32 – 4,092.68) | 8.33 (4) | 0.08 | 3,200 | 4,757.53 (3,426.84 – 6,604.96) |
| **(F_11_)** | 280 | 0.86 ± 0.13 | 2,055.72 (1,297.57 – 3,256.85) | 1.03 (4) | 0.90 | 3,200 | 3,174.30 (2,178.07 – 4,626.19) |

^a^Number of insects tested; ^b^Standard error; ^c^Lethal concentration 50% and confidence interval (CI) at 95%; ^d^Degrees of freedom; ^e^*p* value; ^f^Resistance ratio LC_50_ of the resistant strain/LC_50_ of the susceptible strain and 95% confidence interval.
