# Supplementary material for: Bifenthrin resistance in Dalbulus maidis (Hemiptera: Cicadellidae): inheritance, cross‐resistance, and stability
Source: Pest Manag Sci. 2025 Apr 25;81(8):4810–20. doi: 10.1002/ps.8848 (PMC12268804; doi:10.1002/ps.8848)
Supplement: Supplementary file 2 — Table S2. Concentration‐mortality response (LC50 ± 95% CI) in the absence of selection pressure with bifenthrin during 11 generations in a population of Dalbulus maidis collected in a commercial corn field in Rio Verde, Goiás, Brazil. [file PS-81-4810-s001.docx]

**Table S2.** Concentration-mortality response (LC50 ± 95%CI) in the absence of selection pressure with bifenthrin during eleven generations in a population of *D. maidis* collected in a commercial corn field in Rio Verde, Goiás, Brazil.

| **Strain**  **(generation)** | **n^a^** | **Slope ± SE^b^** | **LC_50_ (95% CI)^c^**  **(μg a.i. ml^-1^)** | **χ² (d.f.)^d^** | ***p*^e^** | **RR_50_^f^** |
| --- | --- | --- | --- | --- | --- | --- |
| Sus | 272 | 1.88 ± 0.21 | 0.64 (0.51 – 0.81) | 5.26 (5) | 0.38 | - |
| **(F_1_)** | 281 | 1.00 ± 0.14 | 113.61 (76.51 – 168.70) | 3.83 (4) | 0.42 | 175.43 (122.00 – 252.24) |
| **(F_3_)** | 280 | 0.84 ± 0.12 | 129.20 (81.37 – 205.15) | 4.29 (4) | 0.36 | 199.51 (136.95 – 290.66) |
| **(F_4_)** | 280 | 1.40 ± 0.15 | 97.57 (71.31 – 133.50) | 5.30 (4) | 0.25 | 150.66 (110.19 – 206.01) |
| **(F_6_)** | 280 | 0.79 ± 0.12 | 74.53 (44.60 – 124.54) | 6.99 (4) | 0.13 | 115.09 (78.27 – 169.22) |
| **(F_7_)** | 280 | 0.90 ± 0.12 | 76.03 (48.03 – 120.36) | 7.19 (4) | 0.12 | 117.41 (81.68 – 168.76) |
| **(F_8_)** | 280 | 1.03 ± 0.13 | 61.85 (40.57 – 94.29) | 2.42 (4) | 0.65 | 95.15 (67.80 – 134.55) |
| **(F_9_)** | 280 | 1.16 ± 0.13 | 30.41 (21.65 – 42.73) | 4.83 (5) | 0.43 | 46.96 (34.17 – 64.54) |
| **(F_11_)** | 280 | 1.13 ± 0.12 | 10.73 (7.32 – 15.74) | 0.90 (4) | 0.92 | 16.58 (12.05 – 22.81) |

^a^Number of insects tested; ^b^Standard error; ^c^Lethal concentration 50% and confidence interval (CI) at 95%; ^d^Degrees of freedom; ^e^*p* value; ^f^Resistance ratio LC50 of the resistant strain/LC50 of the susceptible strain and 95% confidence interval.
