# Supplementary material for: Bifenthrin resistance in Dalbulus maidis (Hemiptera: Cicadellidae): inheritance, cross‐resistance, and stability
Source: Pest Manag Sci. 2025 Apr 25;81(8):4810–20. doi: 10.1002/ps.8848 (PMC12268804; doi:10.1002/ps.8848)
Supplement: Supplementary file 3 — Table S3. Concentration‐response of the treatments 100R:0S, 80R:20S, 50R:50S, 20R:80S, and 0R:100S of Dalbulus maidis to the insecticide bifenthrin. [file PS-81-4810-s003.docx]

**Table S3**. Concentration-response of the treatments 100R:0S, 80R:20S, 50R:50S, 20R:80S, and 0R:100S of *Dalbulus maidis* to the insecticide bifenthrin.

| **Strain**  **(generation)** | **n^a^** | **Slope ± SE^b^** | **LC_50_ (95% CI)^c^**  **(μg a.i. ml^-1^)** | **χ² (d.f.)^d^** | ***p*^e^** | **RR_50_^f^** |
| --- | --- | --- | --- | --- | --- | --- |
| **F1** |  |  |  |  |  |  |
| 0R:100S | 432 | 1.46 ± 0.12 | 0.75 (0.60 – 0.93) | 6.81 (4) | 0.14 | - |
| 100R:0S | 504 | 1.35 ± 0.12 | 2,929.32 (2,314.19 – 3,707.96) | 5.45 (5) | 0.36 | 3,879.90 (3,022.51 – 4,980.51) |
| 80R:20S | 432 | 1.61 ± 0.13 | 934.94 (768.20 – 1,137.88) | 3.22 (4) | 0.52 | 1,238.34 (977.53 – 1,568.73) |
| 50R:50S | 576 | 0.97 ± 0.07 | 150.35 (113.63 – 198.94) | 8.14 (6) | 0.22 | 199.15 (159.62 – 248.48) |
| 20R:80S | 504 | 0.66 ± 0.06 | 17.03 (11.19 – 25.90) | 1.97 (5) | 0.85 | 22.56 (17.51 – 29.06) |
| **F6** |  |  |  |  |  |  |
| 0R:100S | 490 | 1.44 ± 0.12 | 0.72 (0.59 – 0.88) | 7.78 (5) | 0.16 | - |
| 100R:0S | 420 | 0.91 ± 0.09 | 1,536.93 (1,110.48 – 2,127.14) | 3.98 (4) | 0.40 | 2,122.84 (1,621.97 – 2,778.43) |
| 80R:20S | 490 | 1.07 ± 0.08 | 69.12 (52.32 – 91.31) | 9.18 (5) | 0.10 | 95.47 (76.18 – 119.64) |
| 50R:50S | 490 | 0.79 ± 0.07 | 18.41 (12.76 – 26.54) | 4.42 (5) | 0.49 | 25.43 (20.05 – 32.26) |
| 20R:80S | 420 | 1.00 ± 0.09 | 13.01 (9.49 – 17.82) | 8.23 (4) | 0.08 | 17.97 (14.12 – 22.88) |

^a^Number of insects tested; ^b^Standard error; ^c^Lethal concentration 50% and confidence interval (CI) at 95%; ^d^Degrees of freedom; ^e^*p* value; ^f^Resistance ratio LC_50_ of the resistant strain/LC_50_ of the susceptible strain and 95% confidence interval.
